# A cluster randomized trial of a transition intervention for adolescents with congenital heart disease: rationale and design of the CHAPTER 2 study

**DOI:** 10.1186/s12872-016-0307-2

**Published:** 2016-06-06

**Authors:** Andrew S. Mackie, Gwen R. Rempel, Adrienne H. Kovacs, Miriam Kaufman, Kathryn N. Rankin, Ahlexxi Jelen, Cedric Manlhiot, Samantha J. Anthony, Joyce Magill-Evans, David Nicholas, Renee Sananes, Erwin Oechslin, Dimi Dragieva, Sonila Mustafa, Elina Williams, Michelle Schuh, Brian W. McCrindle

**Affiliations:** Department of Pediatrics, University of Alberta, Edmonton, Alberta Canada; Division of Cardiology, Stollery Children’s Hospital, 4C2 Walter C. Mackenzie Center, 8440-112th St. NW, Edmonton, Alberta T6G 2B7 Canada; Athabasca University, Athabasca, Alberta Canada; University Health Network, Peter Munk Cardiac Centre, Toronto Congenital Cardiac Centre for Adults, and University of Toronto, Toronto, Ontario Canada; The Hospital for Sick Children, Toronto, Ontario Canada; Department of Pediatrics, University of Toronto, Toronto, Ontario Canada; Faculty of Rehabilitation Medicine, University of Alberta, Edmonton, Alberta Canada; Faculty of Social Work, University of Calgary, Calgary, Alberta Canada

**Keywords:** Clinical trial, Adolescents, Congenital heart disease, Education, Continuity of care, Transition

## Abstract

**Background:**

The population of adolescents and young adults with congenital heart disease (CHD) is growing exponentially. These survivors are at risk of late cardiac complications and require lifelong cardiology care. However, there is a paucity of data on how to prepare adolescents to assume responsibility for their health and function within the adult health care system. Evidence-based transition strategies are required.

**Methods:**

The Congenital Heart Adolescents Participating in Transition Evaluation Research (CHAPTER 2) Study is a two-site cluster randomized clinical trial designed to evaluate the efficacy of a nurse-led transition intervention for 16–17 year olds with moderate or complex CHD. The primary endpoint is excess time to adult CHD care, defined as the time interval between the final pediatric cardiology appointment and the first adult CHD appointment, minus the recommended time interval between these appointments. Secondary endpoints include the MyHeart score (CHD knowledge), Transition Readiness Assessment Questionnaire score, and need for catheter or surgical re-intervention. Participants are enrolled in clusters based on week of attendance in the pediatric cardiology clinic. The intervention consists of two one-hour individualized sessions between a cardiology nurse and study participant. Session One focuses on knowledge of the participant’s CHD, review of their cardiac anatomy and prior interventions, and potential late cardiac complications. Session Two focuses on self-management and communication skills through review and discussion of videos and role-play. The study will recruit 120 participants.

**Discussion:**

Many adolescents and young adults experience a gap in care predisposing them to late cardiac complications. The CHAPTER 2 Study will investigate the impact of a nurse-led transition intervention among adolescents with CHD. Fidelity of the intervention is a major focus and priority. This study will build on our experience by (i) enrolling at two tertiary care programs, (ii) including a self-management intervention component, and (iii) evaluating the impact of the intervention on time to ACHD care, a clinically relevant outcome. The results of this study will inform pediatric cardiology programs, patients and policy makers in judging whether a structured intervention program provides clinically meaningful outcomes for adolescents and young adults living with CHD.

**Trial registration:**

ClinicalTrials.gov ID NCT01723332

## Background

Major advances in the management of children with congenital heart disease (CHD) have evolved over the past three decades. As a consequence, over 90 % of these children reach adulthood and the population of adolescents and young adults with CHD is growing exponentially [1].However, this emerging “survivor” population has complex needs. These individuals are at risk of substantial cardiac morbidity [2, 3] and mortality [4, 5] in early-to-mid adult years. Unfortunately, 14–53 % of young adults are not successfully transferred to an adult CHD (ACHD) centre after graduating from a pediatric cardiac centre [6–8], and failure to attend an ACHD clinic results in excess cardiac morbidity [7, 9, 10]. Furthermore, adolescents and young adults with CHD have limited knowledge about their heart [11–13], limiting their ability to communicate confidently with health care providers [14].

At present there is a paucity of outcome data regarding the impact and effectiveness of CHD transition interventions*.* Indeed, there is a lack of transition outcome data for chronic pediatric conditions in general. This speaks to the urgency of developing evidence-based intervention programs that will optimize pediatric to adult health care transition using methods that can be readily adopted by clinical programs. The American Heart Association published a Scientific Statement on the subject of transition in 2011 that emphasized the relevance of this topic to the CHD community and acknowledged the lack of data on transition programming [15].

To address these knowledge gaps our team conducted the Congenital Heart Adolescents Participating in Transition Evaluation Research (CHAPTER 1) Study [16] to evaluate a single-session nurse-led transition education intervention, focusing on CHD knowledge, for youth 15–17 years of age. This study significantly improved patients’ knowledge of their CHD lesion in the intervention group at one month, which was sustained at 6 months. The intervention group also had higher self-management skills at six months, even though self-management skills were not the focus of the intervention. Limitations of this study include enrollment at a single center and use of surrogate outcomes (questionnaires) rather than participant behaviors such as attendance at an ACHD clinic. The current study was designed to build upon our experience with CHAPTER 1, specifically to include a self-management component of the intervention, to document time to first ACHD clinic appointment, and to assess generalizability of the intervention across more than one clinical program.

The primary aim of the CHAPTER 2 study is to determine the impact of a nurse-led intervention on time to first ACHD clinic attendance among youth graduating from one of two quaternary-care pediatric cardiology programs. We hypothesize that the transition intervention in combination with usual care will result in superior timing of the first ACHD clinic attendance compared to usual care alone. Secondary aims are to describe: 1) change in adolescent knowledge of their CHD, 2) change in self-management and self-advocacy skills using validated instruments [17, 18], 3) Incidence of cardiac procedures post enrollment, and 4) the frequency and content of verbal and written (email/text messaging) dialogue between nurse and participant.

## Methods

### Study design

**Fig. 1:**
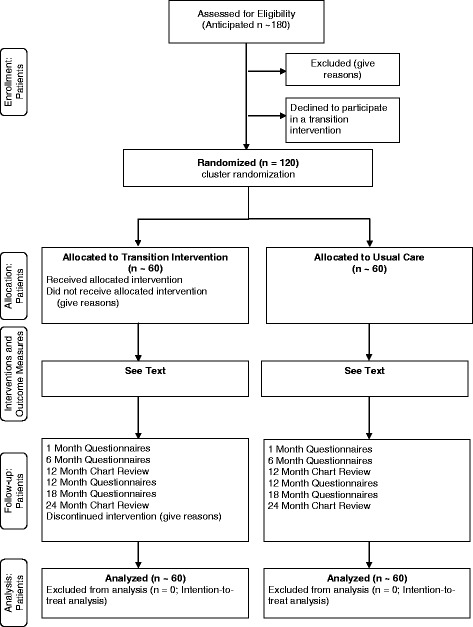
CONSORT diagram

The CHAPTER 2 Study is a two-center cluster randomized controlled trial of a nurse-led transition intervention versus usual care (see “usual care” section below). The intervention will consist of two individualized sessions lasting ~60 min each held two months apart, in or adjacent to the pediatric cardiology clinic. The study is registered with Clinical Trials.gov (ID NCT01723332) and will be conducted in accordance with CONSORT guidelines (Fig. 1) [19].

### Study setting and participants

The study will be conducted at The Hospital for Sick Children (“SickKids”, Toronto, Canada) and the Stollery Children’s Hospital (Edmonton, Canada). These are the largest pediatric cardiology programs in Canada and offer the full range of cardiology and cardiac surgical subspecialty services. We will include 16–17 year olds with moderate or complex CHD (as previously defined) [20] who have not yet been transferred to adult care. Exclusion criteria will be (i) less than a Grade 6 level of reading and comprehension, based on parent report, and (ii) heart transplantation.

### Transition intervention

**Fig. 2:**
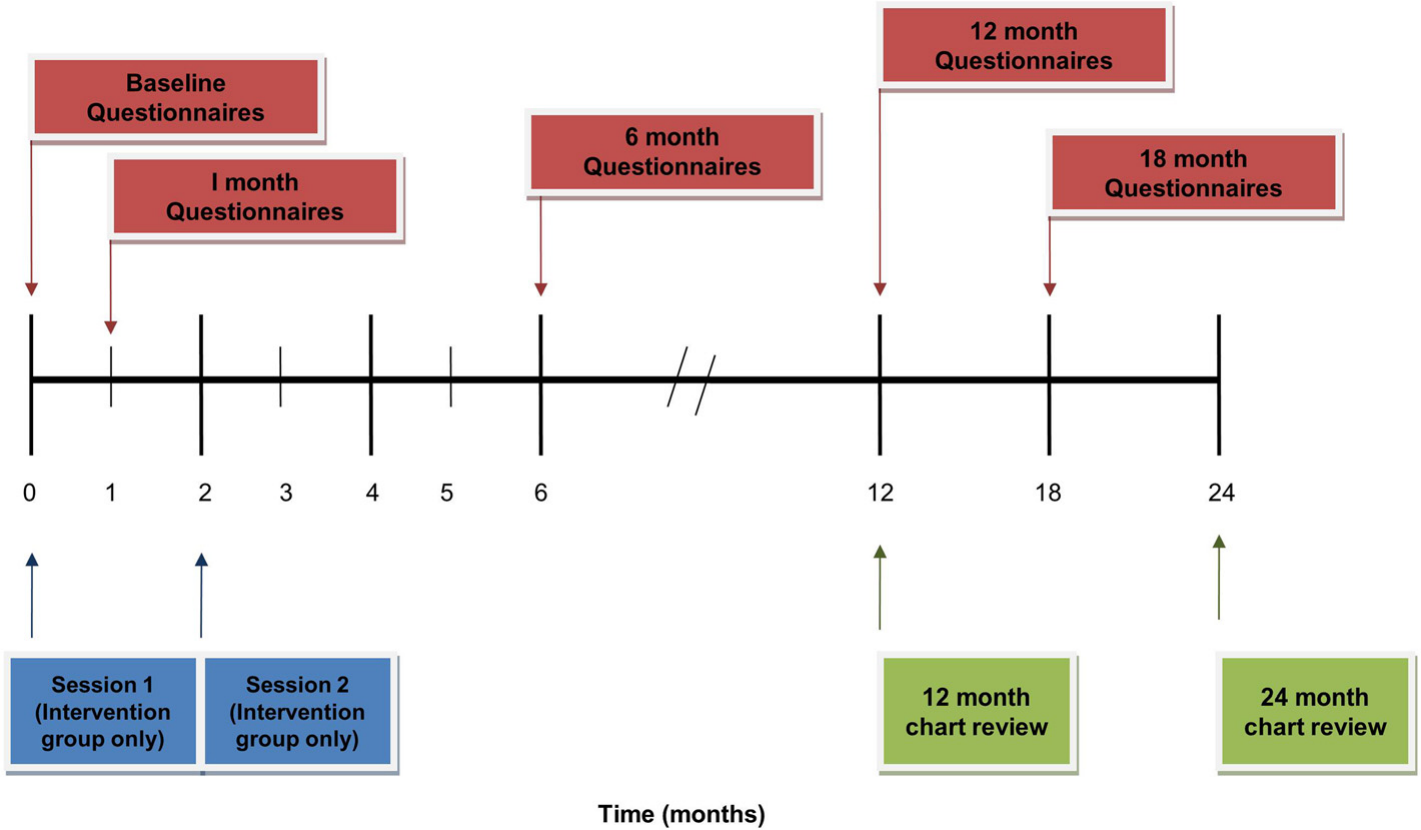
Individual participant timeline

The intervention will be conducted by one of two cardiology registered nurses (RNs) at each site (total 4 RNs) who are experienced working with teens. Youth will attend two one-on-one sessions with the same RN. Sessions will be youth-oriented, interactive, and engaging. Session One will occur immediately after a pediatric cardiology clinic visit to minimize study burden and to adhere with recommendations that transition interventions be delivered in clinical settings [21]. Individual sessions, in contrast to group sessions, allow the content to be patient-specific. Session Two will occur two months after Session One, in conjunction with another clinic appointment or as the sole purpose of a return visit. Individual participant flow through the study is illustrated in Fig. 2.

Immediately prior to Session One, the RN will review the cardiology chart to become familiar with the cardiac history including CHD diagnoses, names and dates of cardiac surgical procedures and cardiac catheterizations, and current cardiac medications and doses. Session One (1 h) will emphasize education; Session Two (1.0–1.5 h) will emphasize self-management. Details of each session are summarized in Table 1.

**Table 1:** Transition Intervention: summary of characteristics and content

Characteristic	Session 1: Emphasis on Education (Held at time of enrollment)	Session 2: Emphasis on Self-management (2 months post Session 1)
Aim	To inform participants about their heart condition	To motivate participants to self-manage and selfadvocate
Approach	Didactic/educational	Experiential/engaging
Role of nurse	Teacher	Facilitator
Mechanism of change	Cognitive/learning	Behavioral/applied action
Content	a) Introduction to transition/its importance	a) Discuss education-related goal set in Session 1
	b) Discussion of confidentiality, promote trust with RN	b) Discussion of “self-management” and its relevance to health
	c) Creation of MyHealth passport, including	c) View & discuss six 1-min videos illustrating interactions between a health care provider (HCP) & young adults with poor vs. strong assertiveness skills
	- name of cardiac condition	
	- previous cardiac interventions	
	- name and purpose of medications	
	d) Review cardiac anatomy (patient-specific)	d) View & discuss “Talking with your doctor” video; review GLADD approach (Give, Listen, Ask, Decide, Do)
	e) Discussion of potential future cardiac complications (patient-specific)	e) Role-play an interaction between a HCP & the participant who practices being assertive
	f) Review names & location of local ACHD cardiologists	f) Discuss SMART goal setting (Specific, Measurable, Attainable, Realistic, Timely)
	g) Introduction to relevant websites	g) Review booklet “When You’re 18”, take home
	h) Discussion of 3 scenarios addressing alcohol, smoking/street drugs, & sexuality/contraception	h) Visit website “Health Care Transitions”
	i) Introduce youth-oriented take-home written materials	i) Encourage email or text messages with RN. Questions posed within 7 days:
		1. “What helped you the most?”
		2. “What helped you the least?”
		3. “Do you have any questions for me?”
	j) Set one education-related goal for session 2	
	k) Accompany participant to the ACHD clinic	
	l) Provide study email address; encourage emails or text messages with RN. Questions posed to participants within 7 days:	
	1. “Where is your MyHealth passport now?”	
	2. “Have you used your MyHealth passport or shown it to anyone?”	
	3. “Do you have any questions for me?”	

*ACHD* adult congenital heart disease; RN: registered nurse

### Treatment fidelity and quality assurance

Consistency of the intervention between study RNs and study participants is a priority of this study. Team education will start with an in-person two-day meeting in Toronto involving study RNs, led by the study investigator (GRR) who co-developed and conducted many of the interventions in the CHAPTER 1 study [16]. Professionally filmed video-tapes depicting Sessions One and Two were prepared for the RNs on how to conduct the sessions with a standardized patient. These teaching videos will be viewed by each study RN and role-playing will be performed for practice.

Audio recordings of sessions One and Two with study participants will be conducted if the participant agrees. Evaluation of treatment fidelity will be based on review of nursing logs and/or audiotapes by other study RNs and team members (ASM, GRR). Monthly conference calls involving all study RNs will be conducted to provide feedback and discuss issues related to the intervention as they arise.

After each session the RN will complete an intervention log. The RN will record completion of each intervention component as described in Table 1, and use field notes to record the participant’s knowledge, enthusiasm, and commitment to the intervention. All intervention logs and field notes will be reviewed monthly by team members (ASM, GRR, and RNs), as part of ongoing quality assurance.

### Usual care

No transition program is formally in place at the Stollery site. Families receive written information about their child’s CHD, suggested timelines for increasing teens’ self-management of his/her CHD, and at the last pediatric visit a “graduation” package with information about the ACHD program is provided. At SickKids, usual care is similar. However, to account for potential differences in usual care, randomization will be stratified by site. With multiple pediatric cardiologists and clinic nurses at each site, clinician-led education is likely variable and constrained by limited clinic time. At both sites, pertinent medical records are sent to ACHD providers at the time of transfer. Both ACHD clinics send a welcome letter to patients indicating approximately when their first ACHD appointment will be. A second letter is sent one month prior to the first ACHD appointment with the date and time. Patients who do not attend are contacted by telephone to reschedule. All components of usual care will be tracked by the project coordinator at each site, and any change in usual care during the study will be documented.

### Group allocation

Participants will be randomized by clusters defined by week of attendance in the pediatric cardiology clinic. As week (not study participant) is the unit of randomization, this is a cluster randomization design. This method of randomization: 1) prevents two adolescents in the same waiting room being allocated to different groups and 2) facilitates scheduling of study RNs, who will be available at short notice to provide the intervention during intervention weeks. Cardiologists will not be informed of group assignment, preventing potential bias by cardiologist co-intervention. We anticipate enrollment of 3–4 patients per week, i.e., cluster size will be ≤ 4. To determine which weeks are “intervention weeks” vs. “usual care weeks”, a biostatistician will prepare a randomization sequence.

A 1:1 ratio of intervention: usual care weeks will be used until midway through enrollment, when there will be a re-evaluation. If there is an imbalance, then there will be an adjustment of the ratio of intervention to usual care weeks.

### Protecting against sources of bias

Participants, their parents, and the study RNs will be aware of group allocation as blinding is not feasible. However, clinical outcomes will be adjudicated by observers who are blind to group allocation (see Secondary outcomes #4 below). Furthermore, participants in both groups will be unaware of the primary outcome (excess time to attendance at first ACHD appointment) and therefore this outcome cannot be consciously influenced, though participants will be aware that the study will track information from the cardiology chart, including the first ACHD clinic visit. Clinic support staff and pediatric cardiologists will be blind to group allocation.

### Outcome measures

Outcome measures correspond to aims as described above. The *primary outcome* will be the excess time between pediatric and ACHD care, defined as the time interval (in months) between the final pediatric visit and the first ACHD visit, minus the recommended time interval between these visits. The “recommended time” interval will be the interval suggested by the cardiologist at the final pediatric visit. For example, if the time between the final pediatric visit and first ACHD visit was 20 months but the pediatric cardiologist recommended this be 12 months, the excess time would be 8 months. If the first ACHD visit takes place before the recommended time, the excess time will be zero. For the rare study participant not having a documented “recommended time”, recommendations for frequency of care will be taken from published guidelines [22, 23]. Excess time to ACHD care is an objective and clinically meaningful outcome, as lapses between pediatric and ACHD care result in increased cardiac morbidity and need for re-intervention [9]. First ACHD appointments that occur 2 or more months later than had been recommended by the referring pediatric cardiologist will result in a review of medical records to identify potential participant or system factors, including a) ACHD waiting list too long to accommodate participant when recommended, b) ACHD team unaware of initial referral, or c) participant did not agree to an earlier appointment.

### Secondary outcomes

Given the complex, multidimensional nature of the transition process, transition interventions need to consider multiple outcomes, including cognitive factors (e.g. CHD knowledge) and self-management behaviours. Therefore, several secondary outcomes are proposed. All baseline questionnaires will be completed in the pediatric clinic, and prior to Session One for those in the intervention arm.

1. Change in MyHeart scores between baseline, 1, 6, 12 and 18 months. The MyHeart scale consists of eight questions that assess participant’s knowledge of their cardiac condition. The MyHeart scale was developed for the CHAPTER 1 study, with a significant improvement in score observed at 1 and 6 months post intervention, compared to participants in the usual care group [16].
2. Change in Transition Readiness Assessment Questionnaire (TRAQ) score between baseline, 1, 6, 12, and 18 months. The TRAQ is the most rigorous transition readiness scale for adolescents. Sawicki et al. identified behaviours relevant to transition, tested item reliability and validity, and then field tested the items with 194 youth with special healthcare needs [18]. Scores range from 0 to 5. Principal component factor analysis revealed two domains explaining 68 % of the total variance: self-management (16 items, mean score 3.01 ± 1.02) assessing skills such as filling prescriptions, understanding treatment side effects, and arranging medical follow-up visits; and self-advocacy (13 items, mean score 3.67 ± 0.77) assessing communication skills with the healthcare team, managing activities of daily living, and use of school and community resources. Internal consistency is high, with Cronbach’s alpha of 0.92 (Self-management) and 0.82 (Self-advocacy). A ceiling effect is unlikely given the mean scores noted above. The reading level is Grade 5.7 and this instrument takes ~5 min to complete. The 12 and 18 month assessments will provide longitudinal data on evolution of self-management and self-advocacy skills and will allow participants in the intervention group sufficient time to apply the skills gained (e.g., to independently book a physician appointment).
3. Change in the Williams’ Scale between baseline, 1, 6, 12, and 18 months. The Williams’ scale is a measure of medical self-management and transition readiness among adolescents with special health care needs, having high internal consistency (Cronbach’s alpha 0.89) [17]. This consists of 21 Likert-scaled items and has a grade 4.9 reading level.
4. Assessment of patient engagement via a cardiologist questionnaire. This questionnaire will be completed by the participant’s primary cardiologist at the first cardiology clinic visit (pediatric or adult site), after month 3 post enrollment. This questionnaire will help determine if those participants in the intervention group were more engaged with their cardiologist then those in the usual care group. The single-page questionnaire consists of six items and takes 1–2 min to complete.
5. Incidence of cardiac re-intervention (surgery or interventional catheterization). Chart review at 12 and 24 months post enrollment will be done independently by two RNs who will be blinded to group allocation. This review will ascertain cardiac hospitalizations and invasive cardiac procedures (since study enrollment), confirm attendance at ACHD clinic, and identify new or progressive complications (e.g., endocarditis, heart failure, stroke, thrombosis, systemic or pulmonary emboli, arrhythmia requiring drug or procedural intervention, renal or hepatic failure) [2]. Discrepant adjudication will be resolved by a cardiologist team member.
6. Frequency and content of verbal and written (email/text messaging/telephone) dialogue between intervention nurse and participant. The intervention RN will complete detailed post-intervention field notes after each session. Telephone communication (phone calls, text messages) and emails will be encouraged from participants at any time; all will be recorded verbatim and analyzed (see Quantitative data analysis below).

### Measurement of outcomes at follow-up

Documentation of first ACHD visit will be done at 12 and 24 month chart review and will include potential barriers to ACHD clinic attendance. Regarding secondary outcomes 1–3, participants will complete the follow-up questionnaires at home, independent of their parent(s). Adolescents not completing the follow-up questionnaires will be contacted by mail, email, or text (depending on their preference) every two weeks for a total of three times, and then telephoned once, to be reminded. A $25 gift certificate will be provided to participants at each of the 1, 6, 12, and 18 month time points to acknowledge their time and commitment to questionnaire completion.

### Proposed sample size

The sample size is based on testing group differences in the primary outcome, excess time to ACHD care, with a two-sided log-rank test. A sample size of 60 patients per group (120 total) will allow detection of a difference between 90 and 70 %, the proportions that have attended their first ACHD clinic appointment by the end of the observation period in the intervention and usual care groups, respectively, with 80 + % power (Type I Error Probability [α] = 0.05), accounting for the “design effect” due to the cluster randomization of the trial. The proportion of 70 % in the usual care group is conservative, as published SickKids data suggests it may be as low as 50 % [8]. If the proportion of usual care participants attending the ACHD clinic is <70 %, the study power will be even higher. The difference between 90 and 70 % is the minimum clinically important difference, as a proportion <90 % attending ACHD care is unacceptably low [15]. Given that the most common reason for a lapse in care prior to first ACHD visit is a belief that cardiac follow-up is not required [9], a misconception that the intervention will address, we believe that a 90 % ACHD clinic attendance in the intervention group is achievable.

### Quantitative data analysis

Intention-to-treat analysis will be used and all statistical tests will be two-sided. Baseline characteristics of the control (usual care) and intervention groups will be summarized using descriptive statistics (e.g., means, medians, standard deviations, frequencies, proportions). Kaplan-Meier plots will display excess time to first ACHD appointment by treatment group. Log-rank tests with a cluster-level bootstrap will test the difference in the excess time distribution between the intervention and control groups. The cluster sizes are likely to be 3–4. All statistical analyses will account for the cluster randomization. Participants who have not attended the ACHD clinic by the end of the study period (i.e., are censored) will still contribute to the primary outcome, as survival analysis is designed to accommodate censored data. We will assess differences in mean scores of each secondary outcome at baseline, 1, 6, 12, and 18 months, where applicable, by treatment group using general linear mixed models [24] that take both the cluster randomization and longitudinal nature of the data into account. Additional analyses will be stratified by a) study site (Edmonton versus Toronto) and b) attended first ACHD appointment (yes/no).

### Qualitative data analysis

All field notes and text/email messages between the RN and participant will be saved, anonymized, and analyzed for codes, categories and subcategories as per qualitative deductive content analysis [25–27]. Data will be analyzed for manifest and latent content, latent content referring to the time that passes between each text message (or email), the word and sentence formations, and the (in)formality and use of language.

## Discussion

The rapidly growing population of adolescent and young adult survivors of CHD is at risk of late cardiac and non-cardiac morbidity and premature mortality. Numerous barriers exist to delivering specialized ACHD care to this population, including a lack of ACHD providers in many developed countries [28, 29]. Unfortunately, few pediatric cardiology programs facilitate the transition of adolescents in their care [30], and many adolescents and young adults experience a gap in care [7, 8, 31] predisposing them to late cardiac complications [9]. To our knowledge, the single-centre CHAPTER 1 study is the only published controlled clinical trial of a transition intervention in the CHD population. The CHAPTER 2 study will build on our experience by (i) enrolling at two tertiary care programs, (ii) including a self-management intervention component, and (iii) evaluating the impact of the intervention on time to ACHD care, a clinically relevant outcome.

### Potential limitations

Several threats to internal validity exist. These include: (i) co-intervention; awareness of the study and of the need for health care transition may influence what cardiologists say to participants, however both groups will benefit equally and this will not create a bias towards one study group. Cardiologists will be unaware of group allocation. (ii) Loss to follow-up; when patients miss appointments, both Edmonton and Toronto ACHD clinics routinely use all available contact information to arrange another appointment and keep patients in care. From the perspective of the study, participants who have not attended the ACHD clinic by the end of the study period will be censored and will still contribute to the primary outcome. (iii) Provider/system factors such as waiting lists may influence the primary outcome. However this will be equally true of participants in both groups. The time interval between the *recommended* date of first ACHD appointment and *scheduled* date of first ACHD appointment will be recorded to capture provider/system factors. The mean wait time for non-urgent referrals to Canadian ACHD clinics is only 4 ± 2 months [29], so we do not anticipate this to be an issue. (iv) Responsiveness of the Williams’ transition readiness scale to intervention is not yet known, though this is just one of six secondary endpoints. Both the TRAQ and MyHeart scale are responsive to an educational intervention [16].

In summary, the CHAPTER 2 study is a two-center cluster randomized clinical trial of a nurse led transition intervention for 16–17 year olds with moderate or complex CHD. Fidelity of the intervention is a major focus and priority. We will evaluate time to first ACHD appointment, CHD knowledge, transition readiness, need for catheter and surgical re-intervention, and qualitative outcomes. The results of this study will inform pediatric cardiology programs, patients and policy makers in judging whether a structured intervention program provides clinically meaningful outcomes for adolescents and young adults living with CHD.

## Abbreviations

ACHD, adult congenital heart disease; CHAPTER, congenital heart adolescents participating in transition evaluation research; CHD, congenital heart disease; RN, research nurse; TRAQ, transition readiness assessment questionnaire
